# Identification of promising sweet sorghum advanced lines for bioenergy production under Mediterranean conditions

**DOI:** 10.1186/s12870-026-08764-8

**Published:** 2026-04-21

**Authors:** Birgul Guden

**Affiliations:** Department of Field Crops, Faculty of Agriculture, Akdeniz University, Antalya, Türkiye

**Keywords:** Biomass, Brix, Juice content, Plant height

## Abstract

**Background:**

Sweet sorghum (*Sorghum bicolor* L. Moench) is one of the most important renewable bioenergy crops that have high biomass production capacity and high sugar content in stems. The aim of this study was to identify promising lines derived from a recombinant inbred population with greater potential for bioenergy in Mediterranean conditions through an initial, line-level evaluation under the target environment.

**Results:**

Twenty-four genotypes derived from a recombinant inbred line (RIL) population were evaluated during the growing seasons of 2021 and 2022 under Mediterranean field conditions. These lines were derived from crossing “Erdurmus” (sweet sorghum) with “Ogretmenoglu” (grain sorghum). There was significant genotypic variation for all bioenergy-related traits, including plant height (PH), stem diameter (SD), fresh biomass yield (FBY), stem yield (SY), Brix (BX), and plant juice content (JC). Heritability varied from 0.17 for JC to 0.91 for PH. The high values for PH and BX indicate strong genetic effects, and the positive correlations between PH, FBY, and SY show that these traits can be beneficial for selection when evaluated in combination. Principal component and cluster analyses classified genotypes that had greater potential for producing more biomass and sugar and revealed distinct trait-based groupings among lines. Several lines, particularly Srg174, Srg334, and Srg351, performed better than the check cultivars for one or more bioenergy-related traits under the conditions of this study.

**Conclusion:**

These lines can be helpful for breeding because they can be used for developing high-yielding cultivars with promising performance under Mediterranean conditions for sustainable bioenergy production and represent valuable genetic material for further advancement in bioenergy-oriented sorghum breeding programs.

## Background

Among the various energy crops, sorghum (*Sorghum bicolor* L. Moench), especially the sweet type, is one of the most valuable sources for renewable bioenergy. This crop is characterized by high biomass capacity, rich sugar content in the stems, and high adaptability in harsh conditions [1] including limited water sources, high temperature, and nutrient-deficient soil environments [2]. These characters demonstrate that sweet sorghum is a promising energy crop for the production of sustainable biofuels and bio-based industries [3].

In sweet sorghum, plant height, Brix, juice content, stem diameter, fresh biomass yield and stem yield are among the important components for bioenergy production [4–6]. The plant height has a strong relationship with vegetative production, and lines that had taller height generally yielded more stem and leaf material. This advantage leads to increased yield of fresh biomass and stem in sorghum [4, 7]. The Brix and plant juice content of sorghum provide valuable information for the quality of stalks; Brix refers to the concentration of soluble sugars, and juice content defines information about the amount of liquid that can be obtained from the plant [8, 9]. These traits directly impact on the quantity of ethanol that can be produced from sweet sorghum. Variations in bioenergy yield among the sweet sorghum lines related not only to these agronomic traits but also to differences in environmental conditions and the genetic structure of each genotype [10]. Variations in temperature, moisture availability, soil fertility, and photoperiod interact with genotypic factors to affect the expression of height, juice quality, and biomass traits. This demonstrates the significance of assessing various genotypes to determine superior one within specific conditions [11].

Low rainfall often reduces the growth and yield of many agricultural crops [12]. Biomass production and sugar development in most species can be restricted under these dry phases with high temperatures in Mediterranean regions [13]. Sweet sorghum, however, generally represents comparatively good performance under such stresses. It can maintain growth and continue producing stalk biomass and stem sugars even when water availability is restricted and temperatures are high, making it a promising option for bioenergy use in these environments [13]. Therefore, it is important to evaluate various sorghum genotypes and identify those that show high performance in specific conditions characterized by frequent high temperatures and drought [14]. Identifying lines that tolerate these conditions while ensuring reliable yields is a major step toward developing cultivars that can contribute to sustainable bioenergy production in Mediterranean areas [6].

As a bioenergy crop, sweet sorghum has been extensively studied in different agro-ecological environments, but research especially conducted under Mediterranean conditions is still limited. The majority of research has focused on hybrid varieties or genetically heterogeneous populations, whereas only a few studies have evaluated the performance of advanced inbred lines derived from recombinant populations (recombinant inbred lines, RILs) under Mediterranean environments [5, 6]. Such lines are ideal for this type of evaluation because they combine genetic diversity at the population level with a high degree of genetic uniformity within each line. Although all RILs originate from a single biparental cross, genetic diversity is maintained through the segregation and independent fixation of parental alleles during successive generations of selfing. As a result, each line carries a unique and stable combination of alleles. Considering that each line is nearly homozygous and genetically stable, the influence of the genotype can be studied more reliably, and the environmental impact can be identified more easily, allowing a clearer separation of genetic and environmental effects. When these lines are derived from parents that contrast strongly in bioenergy-related traits, they can provide valuable insights into the genetic basis and phenotypic variation of these traits, as the recombinant population captures a broad range of trait variation derived from the parental genotypes. Therefore, the present study aimed to evaluate sweet sorghum recombinant inbred lines under Mediterranean growing conditions, with the objective of characterizing their performance for key bioenergy-related traits.

## Materials and methods

### Hybridization and advancement of genetic materials

In 2016, a cross between Erdurmus and Ogretmenoglu sorghum cultivars was performed in the greenhouse of Akdeniz University. The female parent, Erdurmus, is a sweet sorghum cultivar characterized by tall plant height, high Brix value, and high biomass production, while the male parent, Ogretmenoglu, is a grain sorghum cultivar with high grain yield potential. Erdurmus and Ogretmenoglu cultivars were obtained from the Western Mediterranean Agricultural Research Institute (WMARI) situated in Antalya, Türkiye. Both cultivars were officially developed and registered as part of the sorghum breeding program at WMARI, with the breeder seed material provided to Akdeniz University for research purposes.

In 2017, F₁ plants were self-pollinated in the experimental fields of the Department of Field Crops, Faculty of Agriculture, Akdeniz University (36°53′ N, 38°30′ E; altitude 15 m). In the following year, the F₂ population was grown at the WMARI experimental area (36°52′ N, 30°50′ E; elevation 41 m above sea level, Antalya, Türkiye). All F₂ plants germinated from the sown seeds. Among these, twenty-four individual plants (lines) were evaluated for bioenergy-related traits, including plant height, Brix value, juice yield, and fresh biomass production. These plants were selected to represent phenotypic diversity for bioenergy-related traits rather than through intentional or directional breeding selection. These plants were individually self-pollinated using bagging and harvested separately. The selected F₂ plants were advanced to the F₃ generation through progeny-row advancement, and subsequently to the F₄ and F₅ generations by continued self-pollination without intentional or directional selection, with the aim of achieving genetic fixation and line uniformity.

### Experimental site and design

Field experiments were conducted during the 2021 and 2022 growing seasons at the experimental fields of the Department of Field Crops, Faculty of Agriculture, Akdeniz University. The study included twenty-four advanced inbred lines, along with two registered sweet sorghum cultivars, Erdurmus and Uzun (developed by WMARI), used as checks. The experiments were arranged in a randomized complete block design with three replicates for each year.

**Fig. 1:**
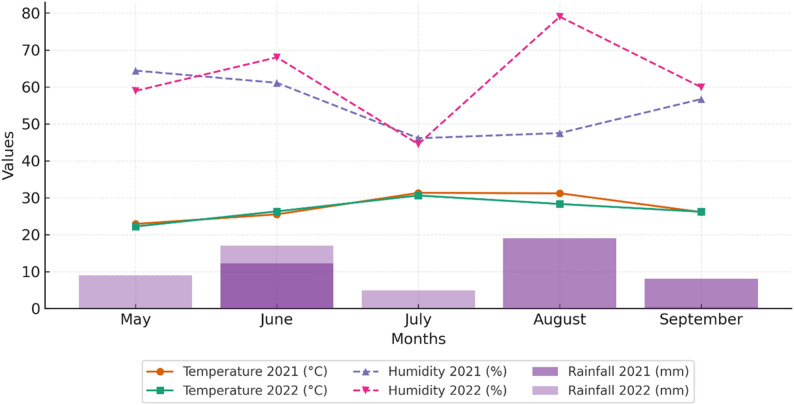
Monthly temperature, rainfall, and relative humidity during the 2021 and 2022 growing seasons at the experimental site (Antalya, Türkiye)

Each plot consisted of four rows, 5 m in length, with 0.70 and 0.20 m were row and plant spacing, respectively. Experimental site soil was classified as loam–clay with slightly alkaline reaction (pH = 7.7) and low organic matter content (1.23%). Total rainfall during the 2021 and 2022 growing seasons were 39.3 mm and 31.4 mm, respectively. The average monthly temperature and relative humidity were 27.4 °C and 55.2% in 2021, and 26.7 °C and 62.1% in 2022, respectively, indicating slightly higher humidity and lower temperature in 2022 (Fig. 1).

### Data collection and trait measurements

From each plot, three representative plants from the two central rows were randomly selected for measurements (PH, SD, and BX). In addition, the two central rows in each plot were harvested, and yield-related traits were recorded from these rows. The following traits were recorded in both years:

- Plant height (PH, cm): measured from the soil surface to the tip of the panicle after full emergence.
- Fresh biomass yield (FBY, t ha⁻¹): determined by cutting the whole plant above ground level, weighing immediately, and converting to per-hectare.
- Stem yield (SY, t ha⁻¹): determined after removing panicles and leaves; measured as the weight of stalks per hectare.
- Stem diameter (SD, cm): measured at the third internode from the base of the main stem using a digital caliper (Clockwise Tools DCLR-0605 Electronic Digital Caliper).
- Plant juice content (PJC, %): was identified by extracting juice from stalks using a roller mill, and the proportion of the extracted juice in the fresh stalk biomass was calculated as a percentage.
- Brix (BX, %): was measured with a digital refractometer (Hanna Instruments, HI96801, USA).

### Statistical analysis

Data analysis was performed using R (v4.3.2) and RStudio. For each evaluated trait, a linear mixed model was fitted to estimate the best linear unbiased predictors (BLUPs). In the model, genotype ${g}_{i}$was considered a random effect, whereas year ${y}_{j}$and replication within year ${r}_{k\left(j\right)}\:$were treated as fixed effects. The significance of the fixed terms was evaluated using F-tests. The model can be expressed as follows:

$${Y}_{ijk}=\mu+{y}_{j}+{r}_{k\left(j\right)}+{g}_{i}+{\epsilon}_{ijk}$$

where ${Y}_{ijk}$ is the observed phenotypic value of the ${i}^{th}$ genotype in the ${j}^{th}$ year and ${k}^{th}$ replication, $\mu$ is the overall mean, ${y}_{j}$ is the fixed effect of the ${j}^{th}$ year, ${r}_{k\left(j\right)}$ is the fixed effect of the replication nested within the year, ${g}_{i}$ is the random effect of the ${i}^{th}$ genotype (${g}_{i}\sim{N}(0,{\sigma}_{g}^{2})$), and ${\epsilon}_{ijk}$ is the random residual error ${(\epsilon}_{ijk}\sim{N}(0,{\sigma}_{e}^{2})$).

Broad-sense heritability (H²) was estimated based on the variance components from the mixed model using the following formula:

$${H}^{2}=\frac{{\sigma}_{g}^{2}}{{\sigma}_{g}^{2}+\frac{{\sigma}_{g\times{y}}^{2}}{y}+\frac{{\sigma}_{e}^{2}}{ry}}$$

Here, σ²g represents the genetic variance, σ²g×y the genotype × year interaction variance, σ²e the residual variance, y the number of years, and r the number of replications.

Correlations among BLUPs of the measured traits were calculated using Pearson’s method. Principal component analysis (PCA) was performed on standardized BLUP values to explore relationships among traits and to determine which traits contributed most to total variation. Hierarchical clustering (Ward’s method, Euclidean distance) was also conducted to group genotypes with similar performance.

All figures, including biplots, heatmaps, and dendrograms, were created using R visualization tools.

## Results

### Variation and descriptive statistics of bioenergy-related traits

The combined data from the F₄ and F₅ showed significant differences among the evaluated lines, as summarized in Table 1. SY exhibited the highest CV (28.9%), while BX showed the lowest (9.08%). The mean PH identified 257.23 cm. Average FBY and SY were 31.55 t ha⁻¹ and 26.78 t ha⁻¹, respectively, while mean BX and JC were at 14.96% and 34.11%.

**Table 1:** Mean values of bioenergy-related traits for F₄–F₅ sweet sorghum advanced lines and check cultivars

Genotypes/cultivars	PH (cm)	FBY (t ha⁻¹)	SY (t ha⁻¹)	SD (cm)	BX (%)	JC (%)
Srg18	190.33	24.45	18.24	2.00	12.64	40.34
Srg33	225.50	29.76	28.00	1.62	15.23	34.17
Srg35	267.83	34.05	29.60	1.83	17.65	38.31
Srg37	270.22	34.24	28.43	1.74	15.49	38.63
Srg74	242.50	37.95	32.52	1.98	15.37	36.04
Srg97	230.78	30.86	25.74	1.85	14.61	26.43
Srg147	167.22	20.14	15.38	1.75	10.92	38.62
Srg156	272.72	41.67	37.19	1.98	15.68	37.09
Srg174	243.56	44.98	37.19	1.92	16.85	32.85
Srg203	318.89	30.19	27.62	1.63	13.77	29.91
Srg214	345.39	26.84	24.29	1.77	15.25	33.17
Srg221	207.72	21.33	16.71	1.61	14.87	38.66
Srg225	167.39	22.76	18.71	1.70	13.09	29.74
Srg238	257.50	32.62	26.86	2.03	15.75	30.34
Srg287	254.78	33.48	27.19	2.12	15.22	38.49
Srg289	236.78	26.81	22.14	1.91	15.08	28.35
Srg293	245.67	30.48	24.57	1.88	15.58	35.57
Srg296	268.44	32.67	28.95	1.93	17.04	33.56
Srg302	268.00	35.81	30.33	2.05	16.19	34.04
Srg303	353.61	37.95	30.10	1.99	11.42	27.35
Srg334	204.11	24.05	20.00	1.63	14.35	37.60
Srg351	327.00	38.86	35.95	2.20	17.15	39.83
Srg366	242.06	27.24	22.90	1.93	14.23	32.81
Srg404	265.94	30.52	27.29	1.76	15.46	32.81
Erdurmus	312.61	32.05	27.33	1.73	16.26	33.57
Uzun	301.33	38.62	33.05	2.27	13.94	28.64
Mean	257.23	31.55	26.78	1.88	14.96	34.11
LSD	35.53^***^	13.01^***^	11.75^***^	0.54^NS^	2.06^***^	13.44^NS^
CV (%)	9.11	27.20	28.94	18.06	9.08	26.00

*PH* Plant height, cm, *FBY* Fresh biomass yield, t ha⁻¹, *SY* stem yield, t ha⁻¹, *SD* Stem diameter, cm, *BX* Brix, %, *JC* Juice content, %

Probability level; ^***^p < 0.001, NS; non-significant

### Performance of lines for bioenergy-related traits

Across two years, the F₄–F₅ lines and check cultivars exhibited wide variation in bioenergy-related traits (Table 1). Significant differences (*p* ≤ 0.001), among lines were detected for plant height (PH), fresh biomass yield (FBY), stem yield (SY), and Brix (BX), whereas no significant differences were observed for stem diameter (SD) and juice content (JC). PH showed a wide range among the lines, from 167.22 cm in Srg147 to 353.6 cm in Srg303. FBY ranged from 20.14 to 44.98 t ha⁻¹, with the highest value observed in Srg174 (44.98 t ha⁻¹), followed by Srg156 (41.67 t ha⁻¹), and the lowest value recorded in Srg147. The highest SY was identified in Srg156 and Srg174 (37.19 t ha⁻¹), followed by Srg351 (36.0 t ha⁻¹); the lowest was in Srg147 (15.38 t ha⁻¹). A narrow variation was observed for SD, ranging between 1.61 (Srg221) and 2.20 cm (Srg351). BX ranged from 10.92 to 17.65%, with the highest value observed in Srg35 (17.65%), followed by Srg351 (17.15%) and Srg296 (17.04%) while the lowest value was recorded in Srg147 (10.9%). The highest JC value was determined in Srg18 (40.34%) while the lowest value was in Srg97 (26.43%). Among the check cultivars, Erdurmus had a relatively high BX (16.26%) with moderate FBY, whereas Uzun had higher FBY (38.62 t ha⁻¹) and SY (33.05 t ha⁻¹).

### Variance components and heritability

Genotypic variance (σ²g) was significant only for PH and BX, whereas it was not significant for FBY, SY, SD, or JC (Table 2). PH showed the highest genotypic variance (σ²g = 2198.94), explaining 73.31% of the total variance. Genotypic variation for FBY and SY was lower (σ²g = 20.75 and 19.89, respectively), explaining 19.22% and 21.98% of the total variance. The genotype × year (σ²g×Y) component varied depending on the trait; the highest value was observed for PH (σ²g×Y = 251.14) representing 8.37% of the total variance, whereas SD showed no detectable interaction effect. This component, σ²g×Y, was also significant for PH and BX but non-significant for the remaining traits; for BX, the genotype × year interaction explained 17.53% of the total variance, while it was negligible for SD (0.00%).

**Table 2:** Variance components and broad-sense heritability (H²) of evaluated traits

Trait	σ²g	σ²g×Y	σ²e	σ²g (%)	σ²g×Y (%)	σ²e (%)	H²
PH (cm)	2198.94^**^	251.14^**^	549.31	73.31	8.37	18.31	0.91
FBY (t ha⁻¹)	20.75^NS^	13.57^NS^	73.65	19.22	12.57	68.21	0.52
SY (t ha⁻¹)	19.89^NS^	10.53^NS^	60.07	21.98	11.64	66.38	0.57
SD (cm)	0.013^NS^	0^NS^	0.115	10.16	0.00	89.84	0.40
BX (%)	1.96^**^	0.81^**^	1.85	42.42	17.53	40.04	0.73
JC (%)	2.86^NS^	1.78^NS^	78.68	3.43	2.14	94.43	0.17

Proportional contribution (%) of variance components was calculated as the ratio of each variance component to the total variance

*σ²g*  genotypic variance, *σ²g×Y*  genotype × year interaction variance, *σ²e*  residual error variance, *H*² broad-sense heritability, *PH* Plant height, cm, *FBY* Fresh biomass yield, t ha⁻¹, *SY* stem yield, t ha⁻¹, *SD* Stem diameter, cm, *BX* Brix, %, *JC* Juice content, %

Probability level; ^***^p < 0.001, NS; non-significant

Broad-sense heritability (H²) ranged from 0.17 for JC to 0.91 for PH. Heritability was high for PH (0.91) and BX (0.73), and genotypic differences for these traits were generally consistent across years, as genotypic variance accounted for a large proportion of the total variance (73.31% for PH and 42.42% for BX) compared with residual variance. These heritability estimates therefore reflect both genotypic effects and the magnitude of genotype × year interaction, rather than representing single-environment values. FBY (0.52) and SY (0.57) showed moderate heritability, with genotypic performance varying among environments, which was reflected by the dominance of residual variance, explaining 68.21% and 66.38% of the total variance, respectively. In contrast, heritability was low for SD (0.40) and JC (0.17), indicating a stronger environmental influence and genotype × year effects on these traits; indeed, residual variance explained 89.84% of the total variance for SD and 94.43% for JC.

### Associations among bioenergy-related traits and clustering

**Fig. 2:**
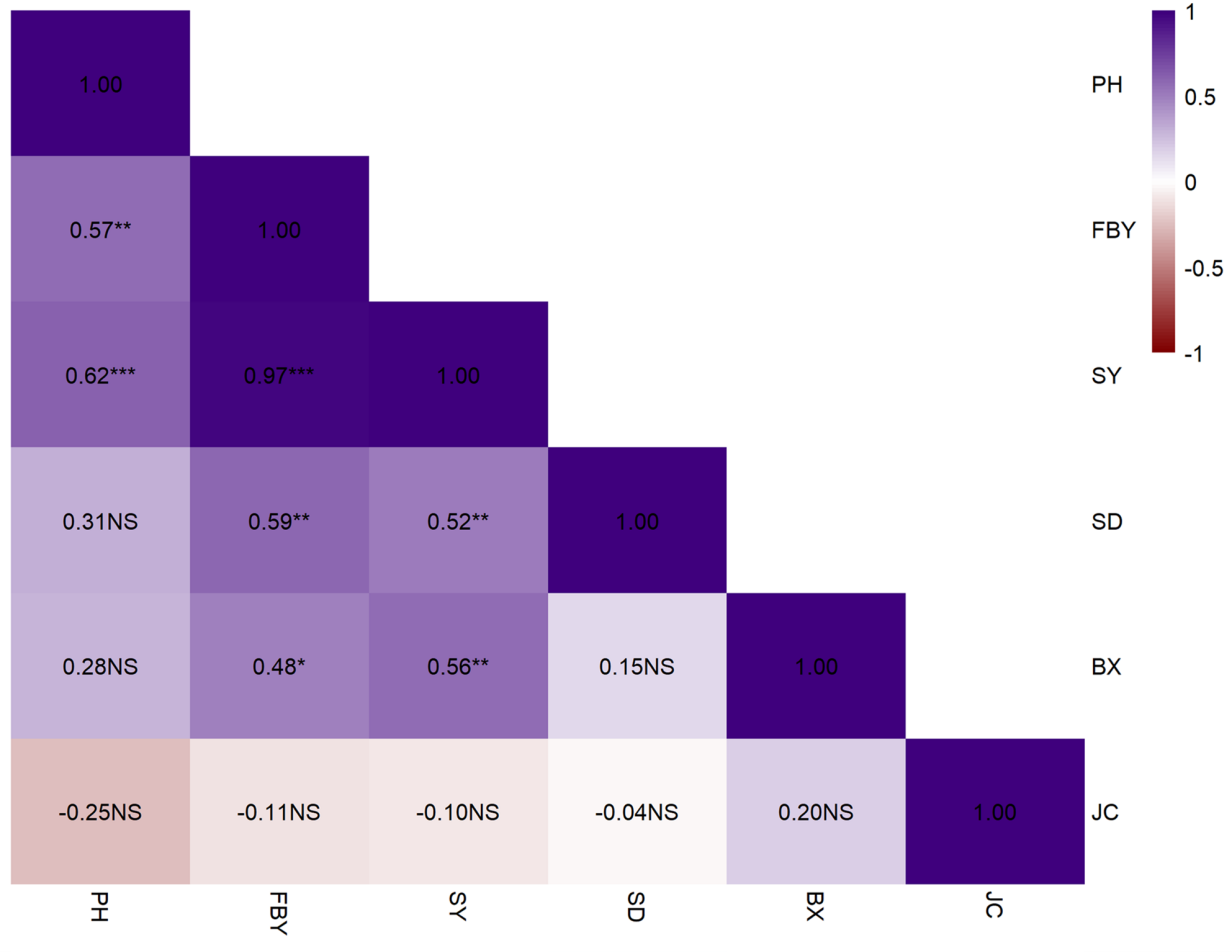
Correlation coefficients among bioenergy-related traits based on combined F₄–F₅ data in sweet sorghum advanced lines PH (Plant height, cm), FBY (Fresh biomass yield, t ha⁻¹), SY (stem yield, t ha⁻¹), SD (Stem diameter, cm), BX (Brix, %), JC (Juice content, %). Probability level; ***p < 0.001, **p < 0.01, NS; non-significant

Correlation analysis showed that several traits in the evaluated lines were associated with each other (Fig. 2). The strongest positive association was determined between SY and FBY (*r* = 0.97, *p* < 0.001). SY was also positively related to PH (*r* = 0.62, *p* < 0.001), while PH was moderately positively correlated with FBY (*r* = 0.57, *p* < 0.01). Moreover, a moderate but positive relationship was determined between SY and BX (*r* = 0.56, *p* < 0.01). In contrast, JC showed weak or negative or non-significant associations with most traits, except for a slight positive related to BX (*r* = 0.20).

**Fig. 3:**
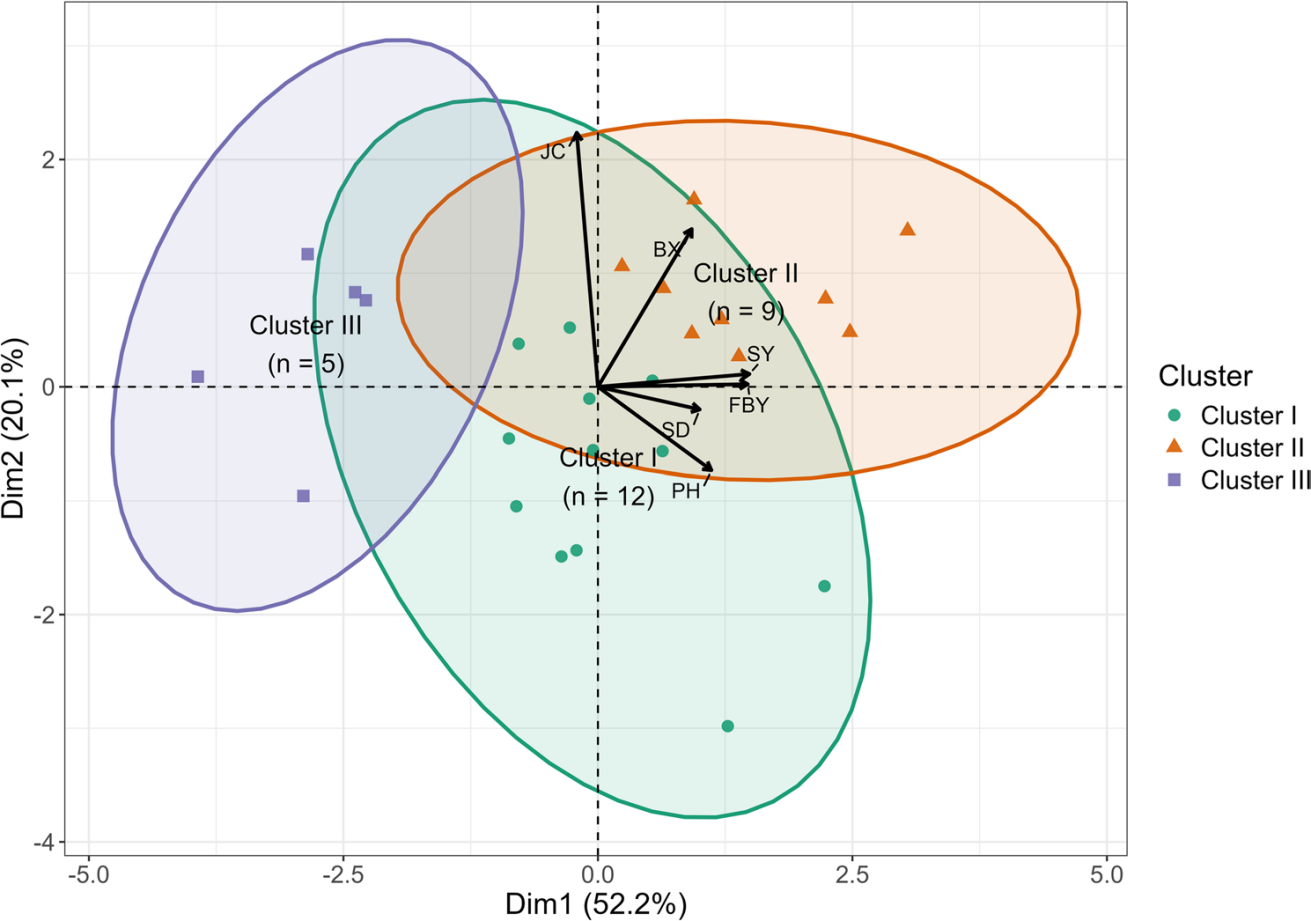
PCA biplot of bioenergy-related traits and clustering of F₄–F₅ sweet sorghum advanced lines PH (Plant height, cm), FBY (Fresh biomass yield, t ha⁻¹), SY (stem yield, t ha⁻¹), SD (Stem diameter, cm), BX (Brix, %), JC (Juice content, %)

Principal component analysis (Fig. 3) based on BLUP values was used to evaluate how traits contributed to the overall variation. The first principal component explained 52.2% of the total variation and was mainly influenced by FBY, SY, PH, and SD, which were positioned close to each other on the biplot. These traits therefore showed a similar response pattern across the evaluated lines. The second principal component explained 20.1% of the variation and largely separated BX and JC from the remaining traits, indicating that quality-related traits followed a different pattern compared with biomass- and yield-related traits. PCA scores separated the lines into three clusters. Cluster II (*n* = 9) was found on the positive side of PC1 and generally showed higher SY, FBY, and BX, suggesting superior biomass productivity combined with higher sugar-related traits. Cluster I (*n* = 12) occurred between the other clusters and displayed a broad spread along PC2, reflecting variation in PH and SD. Cluster III (*n* = 5) was located on the negative PC1 axis and showed lower yield traits.

**Fig. 4:**
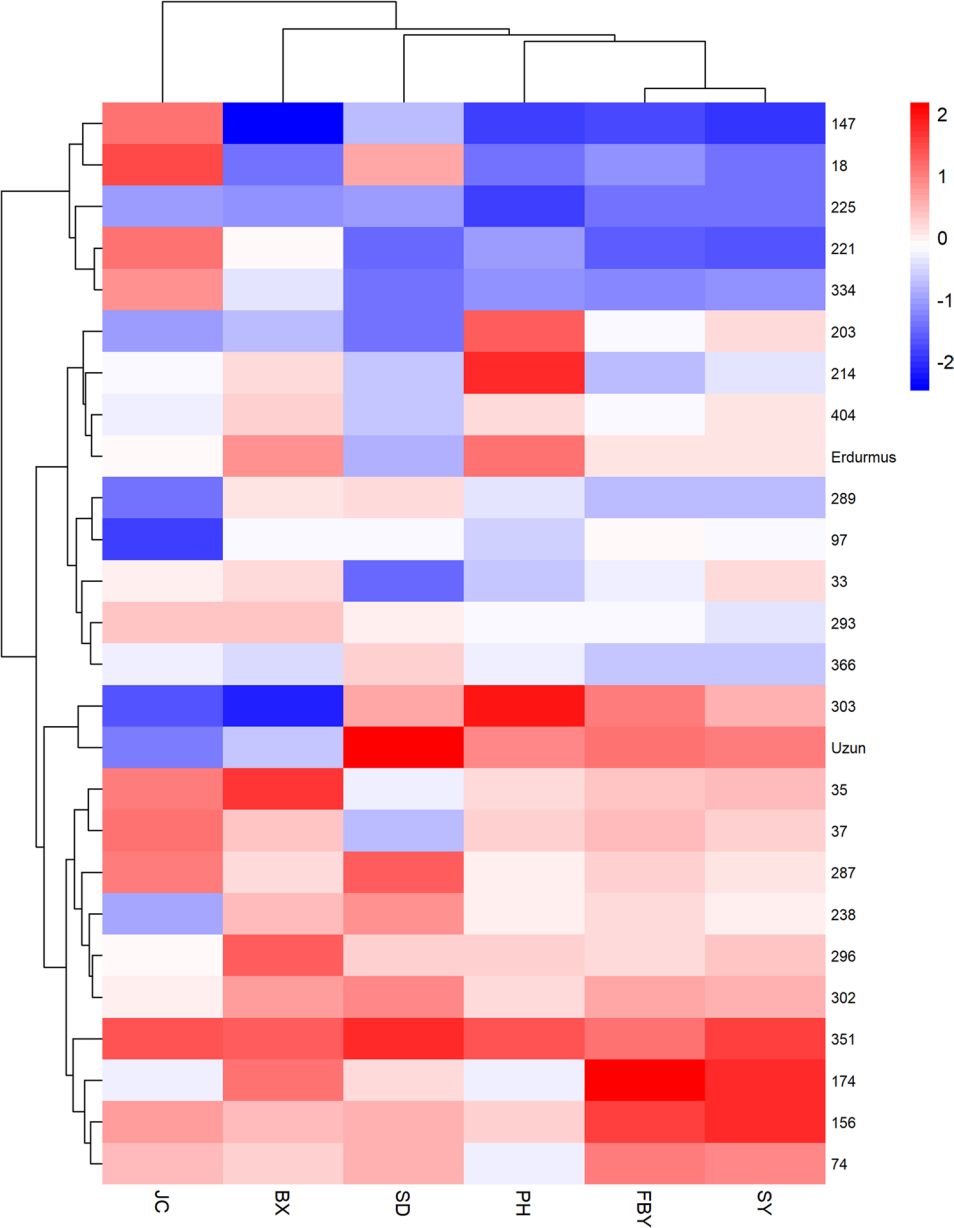
Hierarchical clustering heatmap based on scaled BLUP values of bioenergy-related traits in F₄–F₅ sweet sorghum advanced lines, showing distinct genotype clusters with similar trait profiles Colors represent standardized trait values (blue = low, red = high). Hierarchical clustering grouped the genotypes into distinct clusters based on similar trait profiles, as indicated by the dendrogram

Hierarchical clustering of the scaled BLUP values grouped the lines into three clusters (Fig. 4). Lines with generally higher values for several bioenergy-related traits, such as Erdurmus, Srg334, and Srg174, were found in the same cluster, showing a similar overall performance. By contrast, lines with lower BX or JC values tended to fall into different clusters. The clustering pattern broadly matched the relationships observed in the correlation matrix and the PCA results. Overall, the clusters mainly reflect differences in trait combinations among the RILs.

### Comparison of lines with check cultivars for bioenergy-related traits

**Fig. 5:**
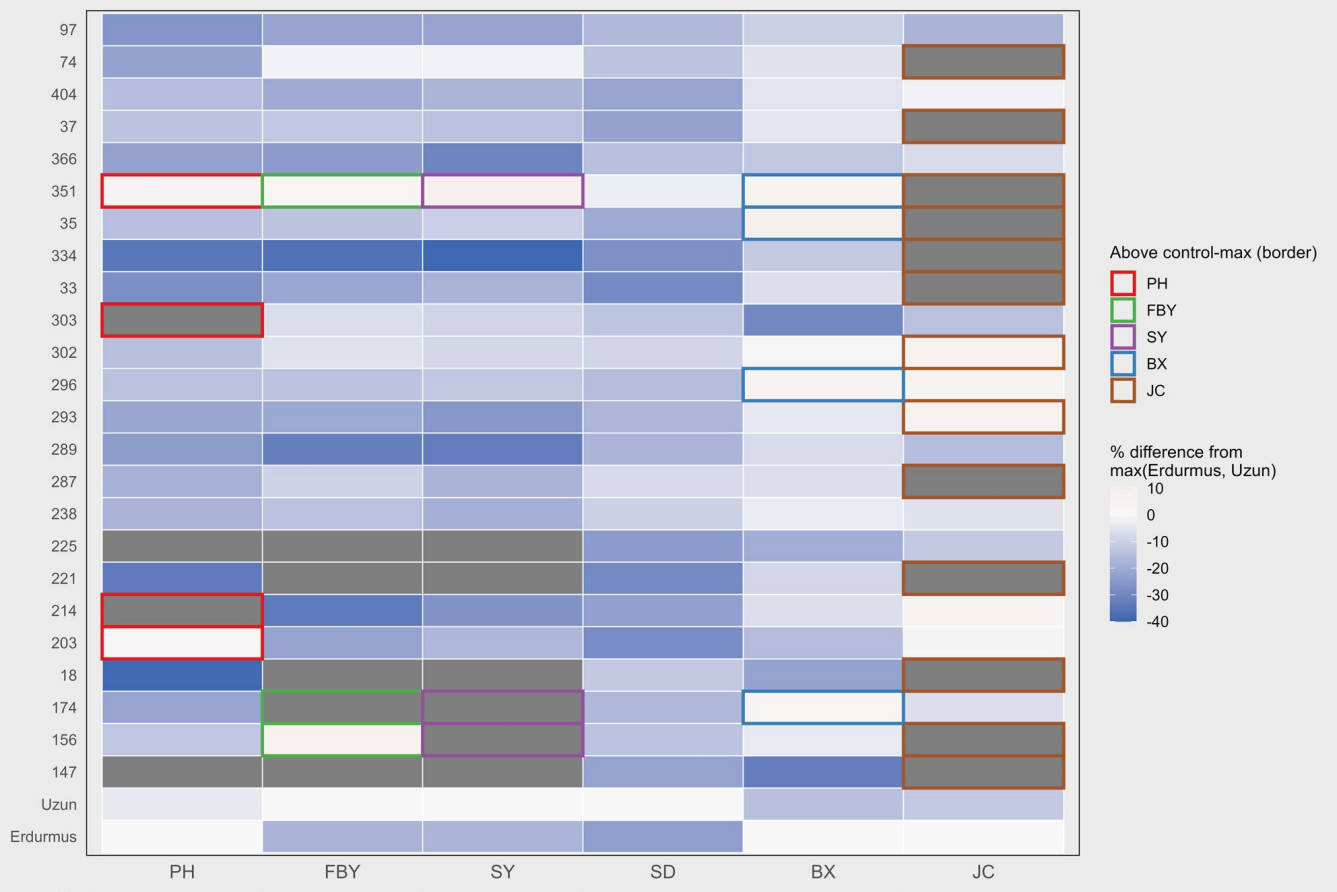
Comparison of advanced lines with check cultivars (Erdurmus and Uzun) for bioenergy-related traits. PH (Plant height, cm), FBY (Fresh biomass yield, t ha⁻¹), SY (stem yield, t ha⁻¹), SD (Stem diameter, cm), BX (Brix, %), JC (Juice content, %)

Several lines performed better than the best control for one or more bioenergy-related traits (Fig. 5). Differences among lines were trait dependent. Some lines exceeded the check cultivars for yield or quality traits. As seen in Fig. 5, these differences were not uniform across traits, and lines that performed well for one trait did not necessarily show similar performance for others. Srg156, Srg174, and Srg351 had higher stem yield (SY). Values reached 37.2, 37.2, and 36.0 t ha⁻¹. This was followed by Uzun, which had a stem yield of 33.1 t ha⁻¹. In the same lines, except for Srg351, fresh biomass yield was above 40 t ha⁻¹. This trend suggests that higher stem yield in these lines was generally accompanied by higher fresh biomass production relative to the control. Srg35 had the highest BX value (17.65%). This value was higher than those of the check cultivars Erdurmus (16.26%) and Uzun (13.94%). Srg18 and Srg174 also showed higher JC values than the checks, reaching 40.3% and 32.85%, respectively. In comparison, JC values of Erdurmus and Uzun were 33.57% and 28.64%. In contrast, Srg147 and Srg225 showed lower values than the controls, especially for PH and FBY. The reductions were around 30–40% compared with the check cultivars. In Fig. 5, these lines are generally positioned on the negative side of the comparison.

## Discussion

For higher bioenergy yields in Mediterranean environments, the development of lines adapted to local growing conditions is crucial [5]. In the present study, several lines showed strong performance for the evaluated bioenergy-related traits under the Mediterranean conditions of the experimental site, indicating that evaluation within the target environment can provide useful preliminary insight (Fig. 5) [15]. These outcomes reflect relative performance under the specific conditions of the study and should not be interpreted as evidence of broad or site-specific adaptation [15]. The evaluated material consisted of advanced, near-homozygous lines assessed at the line level rather than as a segregating population, and the results are therefore intended to support further evaluation rather than definitive inference [16]. Because each RIL-derived line represents a genetically fixed genotype, meaningful comparisons among lines remain possible despite the number of lines evaluated.

Within this framework, the results showed that breeding efforts are more effective when priority is directed to lines that are well adapted to specific conditions. However, conclusions regarding adaptation should be interpreted with caution when based on a single location. Achieving wide adaptation is often difficult in regions where climatic and soil conditions vary considerably. For this reason, testing lines directly in the target environment remains important, as it provides a reliable basis for identifying lines with consistent performance under local conditions [17–19]. Accordingly, the present study focused on evaluating line-level performance under Mediterranean conditions as an initial screening step, and additional multi-location testing will be required to further validate performance and adaptation.

Heritability was high for several traits, showing that genetics had a strong effect and that these traits can be improved through selection [20]. In this study, moderate to high heritability observed for some traits such as PH, SY, and BX indicates that these traits are one of the useful criteria for selection to develop bioenergy performance (Table 2). In particular, the high heritability of plant height (H² = 0.91) was mainly associated with a large proportion of genotypic variance (σ²g = 73.31%), whereas the genotype × year interaction contributed only a minor fraction (8.37%), indicating a relatively stable genetic expression across years. Because heritability estimates were derived from data pooled across years, they also reflect the repeatability of trait expression across seasons. These heritability estimates should be interpreted in the context of genotype × environment interaction rather than as single-environment values. Accordingly, moderate to high heritability suggests that genetic effects were sufficiently consistent across years despite environmental variation. By comparison, SD and JC showed more fluctuation between seasons, indicating that their expression was more sensitive to environmental conditions. For this reason, data from different years were combined and analyzed using a mixed-model approach to reduce year-to-year differences. Under this perspective, the observed heritability patterns are in line with earlier findings on bioenergy-related traits in sorghum. Similar associations between biomass- and sugar-related traits have also been reported in earlier sorghum studies [21–23]. In line with our results, previous work has shown that traits such as stem and biomass weight, juice volume, and sugar or ethanol yield tend to display relatively high heritability together with genetic advance [24]. This suggests that these traits can be practically useful when considering indirect selection for improved bioenergy yield in sweet sorghum.

Multivariate evaluations provided information into variation of traits among the evaluated lines. In the PCA biplot (Fig. 3), traits related to biomass, FBY, SY, PH, and SD were located in close proximity, indicating that they responded in a similar pattern across lines. In contrast, BX and JC appeared on different sections of the plot, which suggests that the components contributing to sugar concentration and the amount of extractable juice are not entirely aligned with the factors influencing biomass accumulation [25]. This separation suggests that biomass-related traits and sugar-related traits may be influenced by partly distinct physiological or genetic mechanisms. The differences obtained indicate that the lines were separated, and high biomass does not always coincide with high juice content. Clustering analysis (Fig. 4) had same pattern with the PCA results, further illustrating the extent of phenotypic variation among the lines. The agreement between PCA and clustering analysis strengthens the interpretation that the observed grouping reflects consistent trait associations rather than artifacts of a single analytical method. Several high-performing lines (Srg174, Srg334, and Srg351) were clustered around Erdurmus. This clustering indicates similarity in overall trait profiles under the conditions of the study, rather than identical performance across all measured traits. Their locations in this cluster showed that some lines with high performance in specific traits rather than across all traits. For example, Srg35 had the highest BX, while Srg18 produced more JC. These trait-specific contrasts within clusters highlight that superior performance can be driven by different trait combinations. These results are consistent with Endalamaw and Adugn’s [26] study; ICSV700 had the highest fresh and dry stalk yield but relatively low juice, sugar, and ethanol yields, whereas genotypes such as NTJ2 and SDSL90167 had high sugar and ethanol yields but only moderate biomass, showing that bioenergy-related traits can peak in different genotypes [4, 6]. Together, these findings emphasize that biomass- and sugar-related traits do not necessarily reach their maximum values in the same genotypes, which is an important consideration for selection strategies based on multiple bioenergy components.

Several lines reached and, in some cases, exceeded the values of Erdurmus regarding FBY and SY, while some performed at almost similar levels to or surpassed Uzun (Fig. 5). These results show that recombination between contrasting parents can produce lines with improved traits, and that some lines can perform as well as or better than either parent [21]. The observed transgressive performance points to substantial genetic potential in the population and helps distinguish lines with improved bioenergy-related traits. This response reflects favorable allele combinations expressed under the conditions of the present study. Overall, the evaluated lines appear to provide useful genetic diversity for the improvement of bioenergy performance in sorghum grown under Mediterranean conditions.

## Conclusion

This study showed that the evaluated advanced lines differed widely for bioenergy-related traits, and several lines had high performance under Mediterranean conditions. Some lines produce high biomass, stem yield, or sugar-related values, in some cases reaching levels similar to the check cultivars. These differences reflect the expression of diverse trait combinations among advanced, near-homozygous lines evaluated at the line level. The findings also indicate that evaluating lines directly in the target environment is important. In this context, the observed performance should be interpreted as relative responses under Mediterranean conditions, providing useful guidance for early-stage evaluation. High heritability estimates for many traits suggest that genetic improvement is achievable through selection. Together with the observed transgressive performance, these results point to the presence of favorable allele combinations contributing to improved bioenergy-related traits. Accordingly, the present results represent an initial step toward identifying promising sweet sorghum lines for bioenergy production under Mediterranean conditions. Overall, the population provides useful material for developing sweet sorghum lines with improved bioenergy performance under Mediterranean conditions and represents a valuable genetic resource for continued progress in bioenergy-oriented breeding programs.

## Data Availability

The datasets supporting the results of this article are included in the main manuscript file.

## Abbreviations

RIL: Recombinant inbred lines
PH: Plant height
SD: Stem diameter
FBY: Fresh biomass yield
SY: Stem yield
BX: Brix
JC: Juice content
